# Resting-State EEG Alpha Asymmetry as a Potential Marker of Clinical Features in Parkinson’s Disease

**DOI:** 10.3390/jpm15070291

**Published:** 2025-07-04

**Authors:** Thalita Frigo da Rocha, Valton Costa, Lucas Camargo, Elayne Borges Fernandes, Anna Carolyna Gianlorenço

**Affiliations:** 1 Neurosciences and Neurological Rehabilitation Laboratory, Physical Therapy Department, Federal University of Sao Carlos, Rodovia Washington Luis, km 235, Sao Carlos 13565-905, Brazil; thalitafrigo@estudante.ufscar.br (T.F.d.R.); valtoncosta@estudante.ufscar.br (V.C.); elayne@estudante.ufscar.br (E.B.F.); 2 Spaulding Neuromodulation Center and Center for Clinical Research Learning, Spaulding Rehabilitation Hospital, Harvard Medical School, 1575 Cambridge Street, Cambridge, MA 02139, USA; lcamargo@mgh.harvard.edu

**Keywords:** electroencephalography, EEG oscillations, alpha asymmetry, neurophysiological pattern, Parkinson’s disease

## Abstract

**Background**: Asymmetrical brain oscillations may be characteristic of Parkinson’s disease (PD). We investigated differences in oscillation asymmetry between individuals with PD and healthy controls and explored associations between the asymmetry and clinical features. **Methods**: Clinical and resting-state EEG data from 37 patients and 24 controls were cross-sectionally analyzed. EEG asymmetry indices were calculated for the delta, theta, alpha, and beta frequencies in the frontal, central, and parietal regions. Independent t-tests and linear regression models were employed. **Results**: Patients exhibited lower alpha asymmetry than controls in the parietal region (t(59) = 2.12, *p* = 0.03). In the frontal alpha asymmetry models, there were associations with time since diagnosis (β = −0.042) and attention/orientation (β = 0.061), and with Movement Disorder Society-Unified Parkinson’s Disease Rating Scale (MDS-UPDRSIII)-posture (β = 0.136) and MDS-UPDRSIII-rest-tremor persistence (β = −0.111). In the central alpha model, higher asymmetry was associated with the physical activity levels (International Physical Activity Questionnaire) IPAQ-active (β = 0.646) and IPAQ-very active (β = 0.689), (Timed Up and Go) TUG dual-task cost (β = 0.023), MDS-UPDRSII-freezing (β = 0.238), and being male (β = 0.535). In the parietal alpha asymmetry model, MDS-UPDRSII-gait/balance was inversely associated with alpha asymmetry (β = −0.156), while IPAQ-active (β = −0.247) and being male (β = −0.191) were associated with lower asymmetry. **Conclusions**: Our findings highlight the potential role of alpha asymmetry as a neurophysiological marker of PD’s motor symptoms, mainly rest tremor, gait/balance, freezing, and specific cognitive domains such as attention/orientation. The models stressed the relationship between disease progression and reduced alpha asymmetry. Brazilian Registry of Clinical Trials (RBR-7zjgnrx, 9 June 2022).

## 1. Introduction

Parkinson’s disease (PD) is one of the most common neurodegenerative disorders, affecting approximately 5.8 million people worldwide in 2019, an increase of 81% since 2000 [1]. This condition is characterized by motor symptoms such as resting tremors, bradykinesia, and rigidity, which typically emerge after approximately 80% of dopaminergic neurons are affected, and several non-motor symptoms such as REM sleep behavior disorder (RBD), hyposmia, constipation, depression, anxiety, apathy, cognitive impairment, among others, with some manifesting early in the disease (e.g., hyposmia and RBD), before substantial dopaminergic neurodegeneration occurs [2]. Clinical manifestations usually begin unilaterally, contralateral to neuronal loss, and this asymmetry persists as the disease progresses, potentially influencing the rate of progression [3]. Despite several studies in past decades, the early-stage diagnosis and treatment still have limitations. Therefore, investigating neurophysiological patterns for diagnostic precision and treatment response, alongside identifying factors linked to neuroplasticity that influence the progression of PD, is essential for advancing our understanding of the disease. These efforts are pivotal for developing strategies to modify disease progression, improve patient outcomes, and optimize healthcare delivery.

The pathological characteristic of PD is mainly the continuous loss of dopaminergic neurons in three circuits: (i) the nigrostriatal pathway, which has projections from the substantia nigra to the dorsal striatum, related to motor symptoms; (ii) the mesolimbic pathway, with projections from the ventral tegmental area to the amygdala, nucleus accumbens and the pyriform cortex; and (iii) the mesocortical pathway, with projections from the ventral tegmental area to cortical regions with D4 receptors, critical for cognitive tasks [4]. However, evidence for the pathology onset outside the brain and divergent progression profiles has been established [5]. Furthermore, the natural aging process combined with the neurodegenerative process of PD promotes structural changes in the brain, with important cortical atrophy and reduction of gray matter volume [6]. These changes exacerbate the symptoms of PD, drastically reducing the cognitive and physical performance of those individuals.

The structural changes can also be reflected by neurophysiological measurements such as electroencephalography (EEG) [7]. This technique is widely used in research due to its low cost, non-invasive nature, and ability to record brain electrical activity, which can detect cortical activity associated with motor and cognitive, or affective status, potentially differentiating several health conditions [7,8,9,10]. In that context, EEG asymmetry–especially alpha asymmetry—is a metric that measures the difference between the right and left hemispheres, employed to investigate patterns of psychopathology, cognitive and emotional control, mainly in the frontal and parietal regions [8]. Overall, the reduction of EEG oscillation asymmetry has been associated with age-related neurophysiological changes [11]. This is because the aging process is often accompanied by a decrease in alpha asymmetry, reflecting reduced functional lateralization. Neurodegenerative conditions are expected to accelerate this process due to reduced interhemispheric connectivity, cortical atrophy, and impairment of the corpus callosum [6]. Though the role of alpha asymmetry related to sensorimotor function in PD has not been established so far, EEG studies have indicated that alpha oscillations play an inhibitory role in movement preparation and execution, and that higher resting alpha power is associated with worse motor outcomes [12,13]. Previous research has indicated that EEG metrics can be correlated with disease severity and dopaminergic neuron loss, suggesting that these metrics may leave specific signatures that can potentially be used to track disease patterns or assess response to disease-modifying therapies [14].

However, the applicability of EEG metrics for diagnosing and monitoring PD is still controversial. The use of the EEG asymmetry index has been investigated in psychology, but only a few studies have used these variables in PD [7,9,15]. Our objectives were to assess resting EEG asymmetry patterns (with focus on alpha oscillations) between individuals with PD and healthy controls and further explore associations of this metric with key clinical features of the patients. Based on the literature, we expected reduced alpha asymmetry in PD, which can be associated with cognitive and functional decline.

## 2. Materials and Methods

### 2.1. Study Design

This exploratory cross-sectional study assessed the baseline data of 37 participants with PD and 24 healthy control individuals included from June 2022 to July 2024. The data are part of a clinical trial aiming to compare modalities of exercise on motor and non-motor symptoms of PD. This study was carried out at the Physical Therapy Department of the Federal University of Sao Carlos (UFSCar), Brazil, approved by the Ethics Board of the UFSCar (approval 5,230,655) and registered in the Brazilian Registry of Clinical Trials (ReBEC, protocol RBR-7zjgnrx, 9 June 2022). All participants were informed and gave their signed consent before enrollment in the study. All ethical considerations were respected, as established in the Declaration of Helsinki (2013) and the Brazilian National Health Board standards. In this report, we followed the *Strengthening the Reporting of Observational Studies in Epidemiology* (STROBE) statement and guidelines.

### 2.2. Recruitment and Eligibility Criteria

Participants were recruited on a voluntary basis from the university outpatient clinic and the surrounding community in Sao Carlos (Sao Paulo, Brazil). Potential participants were contacted by phone after responding to the research advertisements. Volunteers for the healthy adult control group were included if they were 40–80 years of age, without neurological conditions, and had a Mini-Mental State Examination (MMSE) score of at least 20 points (the cut-off point for illiterate individuals). For the PD arm of the trial, the participants had to have a clinical diagnosis of PD confirmed by a neurologist (Hoehn and Yahr stages 1–3, no neurological comorbidities, and stable anti-parkinsonian medication use for at least 30 days before starting the trial. Exclusion criteria for both groups included the development of acute/unstable conditions or withdrawal from participation. After consent, baseline data were collected. Of 51 individuals with PD assessed for eligibility, 37 met inclusion criteria. Twenty-four healthy adults were assessed for the control arm of the trial (assessed only at baseline), and all met the inclusion criteria.

### 2.3. Clinical and Demographic Data

General demographic characteristics, such as age, sex assigned at birth, work and civil status, and education, were collected from all individuals and time since diagnosis, onset side, symptom laterality, Hoehn and Yahr PD stage (1–5 H&Y scale), comorbidities, and level of physical activity from the PD participants. They also underwent the following clinical assessments performed in the ON state of the parkinsonian medications (immediately one hour after administration):

Motor and non-motor symptoms: Movement Disorder Society-sponsored Unified Parkinson’s Disease Rating Scale (MDS-UPDRS); its four parts (MDS-UPDRS I-IV) and respective subscales. MDS-UPDRS I assesses the presence and severity of 13 non-motor aspects of PD, whereas MDS-UPDRS II and III assess motor symptoms affecting upper and lower limbs, posture, and gait (e.g., tremor, bradykinesia, instability, freezing of gait, etc.) and their burden on daily life.

Cognitive function: General function was assessed by the Addenbrooke Cognitive Examination-Revised (ACE-R) and its domains Orientation/attention, Memory, Verbal Fluency, Language, and Visuospatial function; Executive function was assessed by the Trail Making Test parts A and B (TMT A/B).

Neuropsychiatric symptoms: Anxiety was assessed by the Beck Anxiety Inventory (BAI), and depression was assessed by the Geriatric Depression Scale (GDS).

Functional motor outcomes: Functional mobility was assessed by the single and the dual-task Timed Up and Go test (TUG, DT-TUG), and manual dexterity was evaluated by the Nine-Hole Peg Test (9HPT). For the first, besides the duration (in seconds) of the single and DT tests, the cost of DT was also calculated as: (TUG-DT-TUG)/TUG × 100. For the 9HPT, the measure was the execution time (in seconds) for the right and left hands.

### 2.4. Neurophysiological Data

Electroencephalography (EEG) data were collected using a 32-channel EEG system (Neuroelectrics Enobio 32, Barcelona, Spain) from 61 individuals. Resting-state EEG was recorded for five minutes with the participants in a silent room with eyes closed and without any cognitive or motor tasks. Data were collected during the ON state of the PD medication.

### 2.5. EEG Preprocessing

We used EEGLab in MATLAB (MATLAB R2023a, The MathWorks Inc., Natick, MA, USA, 2000) for EEG processing. The methodology is described in detail in our EEG preprocessing pipeline standardized method recently published [16], which has also been applied in previous studies. We followed that method to clean up and remove any biological or nonbiological artifacts that could compromise the analyses. Therefore, the EEG signal processing followed the steps described in this sequence: (i) filtering, (ii) downsampling, (iii) artifact correction, (iv) Independent Component Analysis (ICA), (v) re-referencing, and (vi) frequency band calculation. Also, we followed Makoto’s pipeline using the Darbeliai EEGLAB plugin: (i) Band Pass filter of 1 Hz (High Pass) and 50 Hz (Low Pass); (ii) downsampling from 1000 Hz to 250 Hz; (iii) channels were re-referenced by the average of the electrodes.

Criteria for removing channels: (i) flat for longer than three seconds, (ii) showed high-frequency noise greater than two standard deviations, and (iii) showed correlation with neighboring channels less than 0.8 using the Clean_rawdata EEGLAB plugin (v2.2). We performed a visual inspection and rejection of channels containing artifacts prior to performing the ICA and re-referencing. The remaining channels were fed into the Infomax ICA calculation using the Darbeliai plugin. This analysis demonstrates higher efficiency in identifying components, and it successfully identifies artifacts. The ICLabel toolbox was employed to automatically eliminate components associated with heart rate, blinking, muscle noise, and eye movement.

### 2.6. Resting-State EEG Analysis

After the preprocessing steps, we used the pop_spectopo EEGLab function to process the artifact-free data. We used Fast Fourier Transformation with two-second windows, 50% overlap, Hamming Window, frequency resolution of 0.5 Hz, and interpolation. The EEG oscillations analyzed included delta (1–3.9 Hz), theta (4–7.9 Hz), alpha (8–12.9 Hz), and beta (13–29.9 Hz). For each band, we estimated the relative power, defined as the power within a specific frequency range divided by the total power from 1 to 30 Hz. We calculated the average of the channels selected from the frontal (AF3, AF4, F3, F4, F7, F8, Fp1, Fp2, Fz), central (C3, C4, CZ, FC1, FC2, FC5, FC6), and parietal (CP1, CP2, CP5, CP6, P3, P4, P7, P8, Pz) regions, and used multiple electrodes from each region to calculate the asymmetry.

### 2.7. EEG Asymmetry Index

The EEG asymmetry index was calculated from the natural log-transformed relative power differences between the left and right hemispheres in the three regions of interest (ROI), i.e., frontal, central, and parietal. The asymmetry index was estimated by subtracting the value of the left hemisphere from the right hemisphere (e.g., frontal alpha asymmetry index = natural log-transformed relative power of frontal alpha right − natural log-transformed relative power of frontal alpha left), according to Tomarken et al. (1992) [17]. For calculating the asymmetry indices, the electrodes Fz, Cz, and Pz were not included.

### 2.8. Statistical Analyses

Data analysis was conducted using R Software/RStudio version 2024.09.1 + 394 (Positive Software, PBC, Boston, MA, USA). A descriptive analysis was performed for the clinical and demographic variables. Mean and standard deviation (SD) were calculated for continuous data, and absolute values and percentages for categorical and binary variables. Data distribution and normality were assessed through histograms and Shapiro-Wilk tests. To compare baseline characteristics, independent *t*-tests or Fisher’s exact tests were performed. To compare the EEG asymmetry index between groups, independent t-tests were performed. Statistical significance was established as a *p*-value < 0.05.

Pearson’s correlation tests and univariate linear regression were used to assess the linear relationship between EEG asymmetry index values and the clinical and demographic variables as an exploratory step for the subsequent Multivariate Linear Regression Models. First, we performed univariate linear regressions with the alpha asymmetry index as the dependent variable and other clinical and demographic variables as independent variables. We evaluated four linear regression assumptions: linearity, homoscedasticity, independence, and normality. In this step, all the variables with a *p*-value < 0.20 were selected to be tested in the multivariate analysis. To build our final multivariate models for alpha asymmetry, we kept the variables that showed statistical significance and physiological or clinical rationale, adjusted for age, sex assigned at birth, and other relevant variables. Finally, Bonferroni correction for multiple comparisons was applied to decrease the chance of false positives and facilitate interpretation of the results.

## 3. Results

A description of the clinical and demographic characteristics of the sample involved in this study can be observed in Table 1. The PD sample (*n* = 37) was predominantly male (65%), aged 62 (±11) years, H&Y 2.1 (±0.5), and had a mean time since diagnosis of 5.1 (±3.2) years. The control group (*n* = 24) was composed of healthy adults (without PD or other neurological conditions), 13 females (54%) and 11 males (46%), with a mean age of 59 years (±10). They did not report any symptoms of depression and anxiety. No differences were found between the groups regarding age (t(51) = −0.93, *p* = 0.36), sex (X^2^ = 1.45, *p* = 0.23), levels of physical activity, education and work status (Fisher’s exact test, *p* = 0.49, *p* = 0.06 and 0.30, respectively).

### 3.1. Differences Between Groups

**Figure 1:**
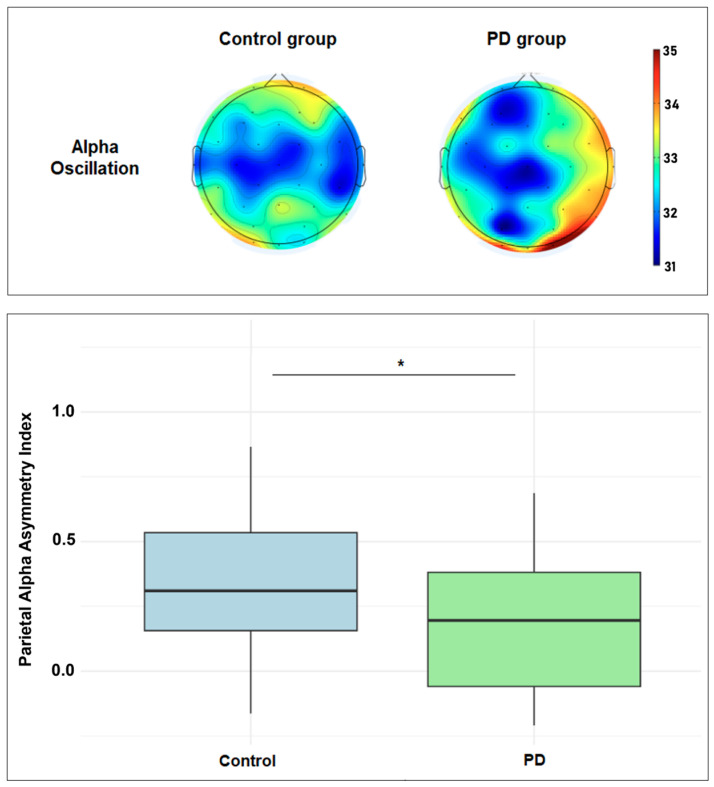
Top figure: scalp plots showing the topographic distribution of the resting-state alpha power (range: 31.0 to 35.0 dB; 10 × log10 P). Bottom figure: boxplot showing the parietal alpha asymmetry index difference between healthy and PD groups. * Statistically significant (*p* < 0.05).

EEG data were obtained from all participants (see Online Resource Supplementary Table S1 for the relative power of all EEG oscillations by group and ROI). The EEG asymmetry index was calculated for all EEG bands—delta, theta, alpha, and beta—across all ROIs. Table 2 compares the two groups for all EEG oscillation asymmetry indices. Independent t-tests indicated that healthy participants showed significantly greater parietal alpha asymmetry (M = 0.37 ± 0.31) than PD participants (M = 0.21 ± 0.26, t(59) = 2.12, *p* = 0.03). A visual representation of the difference is displayed in Figure 1. There were no significant differences between the groups for delta, theta, or beta oscillation asymmetry in the parietal region, nor for any oscillations in the frontal or central regions.

Univariate regression analysis revealed that group (PD vs. control) significantly predicted parietal alpha asymmetry (F(1,59) = 4.89, *p* = 0.031). The PD group showed lower asymmetry than controls (β = −0.160, 95% CI [−0.307, −0.013], *p* = 0.031, R^2^ = 0.076). When adjusted for sex and age, the multivariate model retained significance for group (F(3,57) = 3.42, *p* = 0.023), with PD diagnosis remaining a predictor (β = −0.170, 95% CI [−0.323, −0.018], *p* = 0.030, partial R^2^ = 0.072), although the total explained variance decreased (R^2^ = 0.03).

### 3.2. Correlation Analysis Between EEG Asymmetry and PD Data

**Figure 2:**
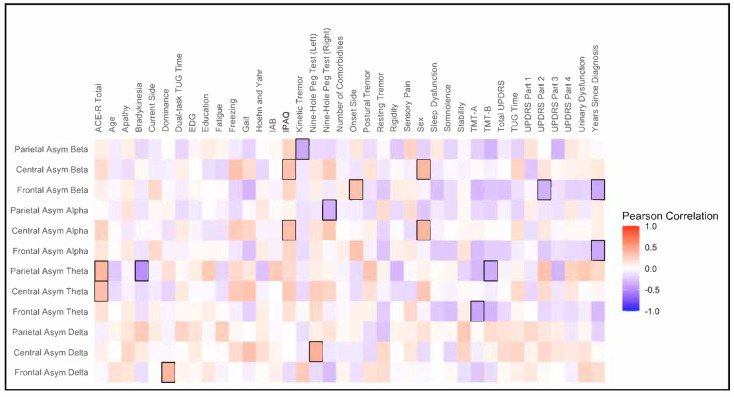
Heatmap of Pearson’s correlation. The *Y*-axis represents the EEG asymmetry index, while the *X*-axis represents clinical and demographic variables. The hot color (“red”) indicates a positive correlation, while the colder color (“blue”) indicates a negative correlation. Statistically significant correlations (*p* < 0.05) are highlighted (black square).

The heatmap shown in Figure 2 illustrates Pearson’s correlations between the EEG asymmetry index and clinical and demographic data. Frontal alpha and beta asymmetry showed a negative correlation with time since diagnosis (r = −0.39, *p* = 0.02; r = −0.38, *p* = 0.03). Central alpha and beta asymmetry demonstrated a positive correlation with the level of physical activity (IPAQ) (r = 0.35, *p* = 0.04; r = 0.35, *p* = 0.04), and central and parietal theta asymmetry showed a positive correlation with cognitive function (ACE-R) (r = 0.35, *p* = 0.04; r = 0.39, *p* = 0.02).

### 3.3. Univariate and Multivariate Models for Alpha Asymmetry in PD

The main clinical and demographic variables potentially associated with alpha asymmetry in the ROIs (*p* < 0.20) can be seen in the Online Resource Supplementary Table S2. The final multivariate models are presented in detail in Table 3, and the adjusted multivariate models can be seen in Supplementary Table S3. Handedness, onset side, and symptom laterality were not significantly associated with the dependent variables nor did they influence the models; therefore, they were not included in the final models. For the frontal alpha asymmetry model, we found two different models. The first model (R^2^ = 0.19) shows, respectively, a negative and a positive association between time since diagnosis (β = −0.038) and attention/orientation (β = 0.060) with frontal alpha asymmetry. In addition to the association with time since diagnosis (β = −0.056), the second model (R^2^ = 0.32) points out, respectively, a positive and a negative association between posture (β = 0.136) and rest tremor persistence during physical assessment (β = −0.098) with frontal alpha asymmetry. Increasing values of posture and rest tremor (subscales of UPDRS III) indicate worse PD outcomes.

For the central alpha asymmetry model (R^2^ = 0.56), both levels of physical activity IPAQ-active (β = 0.639) and IPAQ-very active (β = 0.662), were associated with a higher asymmetry index compared to the irregularly active level. Positive associations were found between the cost of DT-TUG (β = 0.017) and freezing (β = 0.246) with central alpha asymmetry. At the same time, male sex (β = 0.600) was associated with a higher asymmetry index than females. Increasing values of the cost of DT-TUG and rest tremor (subscale of UPDRS III) indicate worse PD outcomes.

For the parietal alpha asymmetry model (R^2^ = 0.34), a negative association was found between the UPDRS II subscale gait and balance (β = −0.127) with alpha asymmetry. Increasing values of this subscale indicate a worse outcome. The active level of physical activity (β = −0.240) and male sex (β = −0.158) were associated with a lower asymmetry index compared to the irregularly active level and the female sex.

## 4. Discussion

This exploratory study aimed to assess the asymmetry of resting EEG oscillations, especially the alpha band, in healthy controls and individuals with PD, and the associations between PD clinical features and brain oscillation asymmetries. We observed lateralized alpha power in the parietal region, with higher right-hemisphere alpha power in the control group, which was significantly lower in the PD group. Additionally, we observed interesting associations between PD clinical and demographic characteristics and alpha asymmetry, which are discussed in the following paragraphs. Although EEG asymmetries, especially alpha asymmetry, have focused mainly on emotional processing, psychopathology, and cognitive control [10,18], we will focus on discussing potential explanations and implications of the results observed in our study.

### 4.1. Differences in Parietal Alpha Asymmetry Between Groups

Alpha asymmetry was lower in the PD group than in the control group. Yet, this difference was found only in the parietal region. Regression analysis showed that the group explained only 6% of the variance in parietal alpha asymmetry, indicating that other factors contribute to the asymmetrical relationship observed in this study. No differences in other brain oscillations were found. The brain is morphologically asymmetrical with a fronto-occipital gradient of cortical thickness asymmetry in healthy brains, which varies depending on sex, age, and handedness, with reductions in that asymmetry being associated with pathological conditions such as autism spectrum disorder and pediatric obsessive-compulsive disorder [11,19]. Pathology in PD manifests with a remarkable asymmetrical characteristic, which translates into the first unilateral identification of motor symptoms [6]. Both microstructural grey matter and functional asymmetries have been identified, differentiating patients with PD from those with PD and cognitive decline and healthy individuals [20,21,22]. Based on that, it can be suggested that the decreased asymmetrical spontaneous alpha activity that we observed may reflect a pathological feature or a compensatory response in PD.

Previous studies have identified a widespread slowing of oscillatory brain activity since the early stage of the disease in non-demented patients with PD, regardless of time since diagnosis, severity, and dopaminomimetic treatments [15]. They also found an association between longer disease duration (years since diagnosis) and reduced low alpha power in the right temporal region, which agrees with our findings. A study aimed to identify neural resource allocation during an odd-ball paradigm found that individuals with PD showed faster response latencies to the task-irrelevant standard tones, slower response latencies to target tones compared to healthy controls, and longer reaction times, suggesting inefficient resource allocation. They displayed reduced parietal alpha activity, associated with distractor suppression and functional inhibition, altered in patients with PD [23]. In the context of emotional processing, differences were found between healthy individuals and those with PD during the processing of emotions [9]. The patients showed lower alpha asymmetry throughout several regions, whereas the healthy controls showed higher right than left hemisphere activation.

Though not all those studies evaluated resting-state EEG or considered asymmetrical differences, our findings may point to potential explanations. For instance, the decreased parietal alpha asymmetry may be a marker of misadjusted functional inhibition and overactivated compensatory mechanisms of lateralized cortical processes (sensorimotor, attentional, perceptive, emotional, cognitive), possibly mediated by the right hemisphere [24,25,26]. These studies also suggested that increased resting-state alpha power in PD may reflect excessive inhibitory control from the thalamocortical system, which contributes to the difficulty initiating movements [12]. In this context, resting asymmetrical alpha oscillation may reflect a compensatory process of modulating and optimizing movement, as some of our models seem to indicate (tremor persistence, freezing, gait/balance, and even higher levels of physical activity).

### 4.2. Frontal Alpha Asymmetry Models

These models present an inverse relationship between time since diagnosis and alpha asymmetry, which suggests that a reduction in the activity of this oscillation in the right hemisphere may follow the progression of the disease, independent of age. Accordingly, attention/orientation showed a positive relationship, meaning increased asymmetry is expected in patients with better cognitive performance in this domain. These findings align with the results of previous studies [15,23,27]. The second model adds two associations: the higher the MDS-UPDRSIII-posture scores (worse outcome), the higher the alpha asymmetry, and the higher the MDS-UPDRSIII-rest tremor persistence scores (worse outcome), the lower the alpha asymmetry. Though these results seem to be contradictory, they have different contexts. Posture alterations tend to increase with aging (the regression analysis shows the modifier effect of age). Thus, it is not an exclusive PD characteristic and relates to what we observed in the healthy control: more asymmetry (though only in the parietal region). However, rest tremor is a proper PD symptom. Thus, it was associated with decreased asymmetry, reinforcing the potential role of reduced alpha asymmetry as a marker of alteration in PD. It suggests that higher alpha asymmetry reflects a better motor adjustment or compensated disease state, which seems to agree with previous evidence showing an association between increased alpha oscillation power with worse motor outcomes in PD [13].

### 4.3. Central Alpha Asymmetry Model

This model highlights the associations between the physical activity levels and alpha asymmetry. It indicates that the individuals with increasing activity levels (compared to irregularly active individuals) exhibit higher alpha asymmetry in the central region. This finding reinforces that alpha asymmetry potentially marks more adjusted brain dynamics and highlights the role of physical activity in keeping it up [28,29,30]. Interestingly, sex (male) was associated with a more notable change in alpha asymmetry, stressing the possible role of sex in explaining the differences in asymmetry. The model also indicated that the cost of a dual task (here, the difference in performance between a single and a dual task) and the impact of freezing on daily activities (MDS-UPDRS II) were positively associated with higher alpha asymmetry. However, when measuring the effect of sex on these variables in the model we verified that sex significantly modifies the associations between freezing and alpha asymmetry, even inverting the direction of the association for females (freezing) and increasing the coefficients for the male sex (freezing and cost of the dual task). This highlights the sex differences in PD manifestation (like the pace of neurodegeneration) and the resulting asymmetrical relationships, which can mediate and confound the results, as stressed in other studies on PD and neuropsychological conditions [6,11,31].

### 4.4. Parietal Alpha Asymmetry Model

This model indicated that deficits in gait/balance (MDS-UPDRS II) are inversely associated with alpha asymmetry (like rest tremor persistence in the frontal model), reinforcing that reduced asymmetry may reflect a worse motor outcome in PD. Different from the previous model, the level of physical activity (active in comparison to irregularly active) and the male sex were associated with lower alpha asymmetry. This inversion may be explained by brain region dynamics; that is, it is possible that physical activity and sex-related covariates may modulate alpha activity differently throughout brain regions.

A correction for multiple comparisons was applied to reduce the risk of false positives and aid interpretation. The alpha central and parietal models remained significant after correction, while some variables in the frontal models no longer showed significant results. However, this should be interpreted with caution, as this study is essentially exploratory, and some effects may be subtle to detect. For example, the independent variables in the frontal models showed small to medium effect sizes for which the statistical power of the analyses may not have been sufficient (around 50%).

### 4.5. Strengths and Limitations

These findings warrant further investigation in future confirmatory studies. The limitations of this study are related to the cross-sectional design and small sample size, though it still provides important insights regarding associations. Given the sample size, the multivariate models had to be limited in the number of independent variables to respect modeling assumptions, which restricted our analysis. Yet, both unadjusted and adjusted models show a robust result with no significant differences, which strengthens the conclusions. Despite those limitations, in this study, we observed a difference in alpha asymmetry between groups and the associations between EEG oscillations asymmetries and relevant clinical outcomes.

## 5. Conclusions

Our findings highlight the potential role of alpha asymmetry as a neurophysiological marker of PD’s clinical manifestations, especially motor symptoms (rest tremor, gait/balance, and freezing) and specific cognitive domains, such as attention/orientation. This study emphasized the association between disease progression and reduced alpha asymmetry and the potential differences in this EEG metric between healthy individuals and those with PD. Future validation of these results must investigate the reliability of using resting-state alpha asymmetry, within-individual dynamics, and temporal test–retest stability. The hypothesis that decreased parietal alpha asymmetry reflects a mark of misadjusted functional inhibition of cortical processes warrants further research, and careful consideration should be given to interhemispheric interactions and effect modifiers such as sex assigned at birth. Such an investigation is important to verify the potential applications of this metric in clinical and research settings.

## Figures and Tables

**Table 1:** Clinical and demographic characteristics of the sample.

Variables	PD (*n* = 37) Mean ± SD or *n* (%)	Control (*n* = 24) Mean ± SD or *n* (%)	*p*-Value
Age	62 ± 11	59 ± 10	0.36
Sex: Female/male	13 (35%)/24 (65%)	13 (54%)/11 (46%)	0.23
Education			0.06
Primary incomplete	2 (5%)	0 (0%)	
Primary to secondary	8 (22%)	0 (0%)	
High school	4 (11%)	3 (13%)	
College (incomplete)	12 (32%)	9 (37%)	
Graduation	11 (30%)	12 (50%)	
Work status			0.30
Retired	23 (62%)	12 (50%)	
Working	13 (35%)	12 (50%)	
Unemployed	1 (3%)	0 (0%)	
Physical activity (IPAQ)			0.49
Sedentary	0 (0%)	2 (8%)	
Irregularly active	14 (38%)	10 (42%)	
Active	15 (41%)	8 (33%)	
Very active	8 (22%)	4 (17%)	
Time since diagnosis (years)	5.1 ± 3.2	NA	-
H&Y stage	2.1 ± 0.5	NA	-
MDS-UPDRS I (non-motor)	11.9 ± 6.6	NA	-
MDS-UPDRS II	10.7 ± 7.2	NA	-
MDS-UPDRS III (motor)	26.7 ± 10.8	NA	-
MDS-UPDRS IV	3.2 ± 3.7	NA	-
MDS-UPDRS total	52.4 ± 19.0	NA	-
Mental status (MMSE)	26.2 ± 2.8	-	-
Depression (GDS)	10.9 ± 3.0	-	-
Anxiety (BAI)	12.8 ± 9.2	-	-

*p*-values from independent t-tests or the Fisher exact test. H&Y, Hoehn and Yahr scale (PD staging); MDS-UPDRS, Movement Disorder Society-Unified Parkinson’s Disease Rating Scale; MMSE, Mini-Mental State Examination; GDS, Geriatric Depression Scale; BAI, Beck Anxiety Inventory; IPAQ, International Physical Activity Questionnaire; NA, not applicable.

**Table 2:** Between-group comparisons of EEG asymmetry indices by ROI.

Asymmetry Index	Control Group *n* = 24		PD Group *n* = 37		
	Mean	SD	Mean	SD	*p*-Value
Frontal					
Delta	−0.01	0.23	−0.04	0.14	0.461
Theta	0.10	0.24	0.05	0.25	0.440
Alpha	0.08	0.34	0.08	0.36	0.968
Beta	0.03	0.45	0.05	0.34	0.849
Central					
Delta	0.06	0.18	−0.02	0.28	0.245
Theta	0.03	0.27	0.06	0.48	0.778
Alpha	−0.02	0.40	0.05	0.60	0.634
Beta	−0.12	0.42	−0.09	0.58	0.801
Parietal					
Delta	0.53	0.56	0.50	0.58	0.846
Theta	−1.54	1.04	−1.10	1.00	0.104
Alpha	0.37	0.31	0.21	0.26	0.033 *
Beta	0.14	0.30	0.15	0.33	0.865

With regard to independent *t*-tests, values are represented as mean and SD. * Statistically significant difference.

**Table 3:** Multivariate models for frontal, central, and parietal alpha asymmetry in PD.

Variables	β-Coefficient [95% CI]	Standard Error	*p*-Value	Bonferroni Correction	Adjusted R^2^
FRONTAL					
Time since diagnosis (years)	−0.038 [−0.072, −0.003]	0.017	0.034	0.101	0.19
Attention/orientation	0.060 [0.004, 0.116]	0.027	0.036	0.107	
Time since diagnosis (years)	−0.056 [−0.089, −0.023]	0.016	0.002	0.006	0.32
UPDRS III_posture	0.136 [0.047, 0.225]	0.044	0.004	0.015	
UPDRS III_rest tremor persistence	−0.098 [−0.192, −0.004]	0.046	0.042	0.168	
CENTRAL					
IPAQ (active)	0.639 [0.336, 0.942]	0.149	0.0002	0.0009	0.56
IPAQ (very active)	0.662 [0.294, 1.031]	0.180	0.0009	0.005	
DT-TUG (cost)	0.017 [0.004, 0.029]	0.006	0.009	0.055	
UPDRS II_freezing	0.246 [0.061, 0.431]	0.091	0.011	0.065	
Sex (male)	0.600 [0.317, 0.883]	0.139	0.0001	0.0009	
PARIETAL					
UPDRS II_gait/balance	−0.127 [−0.224, −0.030]	0.047	0.012	0.058	0.34
IPAQ (active)	−0.240 [−0.399, −0.079]	0.079	0.005	0.023	
Sex (male)	−0.158 [−0.305, −0.010]	0.072	0.037	0.184	

UPDRS, Unified Parkinson’s Disease Rating Scale (Movement Disease Society); IPAQ, International Physical Activity Questionnaire; DT-TUG (cost), the difference (%) between the single and the dual-task Timed Up and Go tests.

## Data Availability

The data that support the findings of this study are available from the corresponding author upon reasonable request due to privacy reasons.

## Supplementary Materials

The following supporting information can be downloaded at: https://www.mdpi.com/article/10.3390/jpm15070291/s1, Table S1: Resting-state EEG relative power (%) by brain oscillations and regions of interest; Table S2: Exploratory univariate analysis (*p* = 0.20). Associations between clinical and demographic variables with alpha asymmetry by ROI; Table S3: Adjusted multivariate models for frontal, central, and parietal alpha asymmetry in PD.

## Abbreviations

The following abbreviations are used in this manuscript:

PD	Parkinson’s Disease
EEG	Electroencephalography
UFSCar	Federal University of Sao Carlos
ReBEC	Brazilian Registry of Clinical Trials
H&Y	Hoehn and Yahr scale
MMSE	Mini-Mental State Examination
MDS-UPDRS	Movement Disorder Society-Sponsored Unified Parkinson’s Disease Rating Scale
ACE-R	Addenbrooke Cognitive Examination
TMT A/B	Trail Making Test parts A and B
GDS	Geriatric Depression Scale
BAI	Beck Anxiety Inventory
TUG	Timed Up and Go
DT-TUG	Dual-Task Timed UP and Go
9HPT	9-Hole Peg Test
ICA	Independent Component Analysis
ROI	Regions of Interest
IPAQ	International Physical Activity Questionnaire
