# Supplementary material for: Resting-State EEG Alpha Asymmetry as a Potential Marker of Clinical Features in Parkinson’s Disease
Source: J Pers Med. 2025 Jul 4;15(7):291. doi: 10.3390/jpm15070291 (PMC12296173; doi:10.3390/jpm15070291)
Supplement: Supplementary file 1 [file jpm-15-00291-s001.zip › jpm-3710541-supplementary.pdf]

Supplementary Table S1. Resting-state EEG relative power (%) by brain oscillations and regions of interest.

**Oscillations**	**Healthy group *n* = 24**			**PD group *n* = 37**		
Frontal	Median	Q1	Q3	Median	Q1	Q3
Delta	56.7	40.8	75.2	49.3	25.5	78.0
Theta	7.2	4.3	9.1	8.6	4.5	12.8
Alpha	6.4	2.8	13.6	8.8	4.5	13.5
Beta	5.9	3.9	10.6	5.8	3.9	10.6
Central						
Delta	55.6	31.3	75.2	48.3	27.7	76.7
Theta	5.7	4.3	9.0	9.1	4.1	12.8
Alpha	6.2	2.6	13.9	8.6	3.2	12.6
Beta	6.2	3.1	11.3	7.0	2.9	10.1
Parietal						
Delta	47.2	33.0	68.1	49.2	30.2	76.3
Theta	6.6	3.6	9.1	9.1	4.3	13.5
Alpha	8.1	3.5	24.9	10.5	4.2	15.9
Beta	6.3	4.0	10.8	6.5	2.8	11.0

Supplementary Table S2. Exploratory univariate analysis (*P* = 0.20). Associations between clinical and demographic variables with alpha asymmetry by ROI.

**Frontal**			
**Variables**	***b*-coefficient**	**SE**	**Unadjusted *P*-value**
Diagnosis (years)	-0.041	0.018	0.030
Attention/orientation	0.065	0.029	0.032
UPDRS II_gait_balance	-0.148	0.076	0.059
Work status (working)	0.225	0.123	0.075
UPDRS III_posture	0.089	0.049	0.077
UPDRS III_gait	-0.133	0.078	0.097
UPDRS III_speech	-0.116	0.070	0.109
UPDRS I_somnolence	-0.083	0.051	0.117
IPAQ (very active)	0.253	0.159	0.120
UPDRS II	-0.013	0.008	0.124
Symptoms side (bilateral)	0.171	0.117	0.153
UPDRS III_rest tremor persistence	-0.076	0.055	0.175
UPDRS I_sleep dysfunction	-0.051	0.038	0.181
TMT A	-0.002	0.002	0.194
UPDRS II_speech	-0.060	0.046	0.201
**Central**			
**Variables**	***b*-coefficient**	**SE**	**Unadjusted P-value**
UPDRS III_rest tremor (LLL)	-0.492	0.169	0.006
IPAQ (active)	0.566	0.205	0.009
Sex (male)	0.495	0.191	0.014
UPDRS III_rest tremor (RLL)	-0.927	0.411	0.030
UPDRS I_halucinations	-0.330	0.158	0.043
DT-TUG (cost)	0.017	0.008	0.053
IPAQ (very active)	0.445	0.244	0.078
UPDRS II_freezing	0.229	0.126	0.079
Nine Hole Peg Test (right)	0.015	0.008	0.084
Work status (working)	0.361	0.204	0.085
UPDRS III_agility (RLL)	-0.247	0.142	0.090

UPDRS III_arm pronosupination (left)	0.178	0.104	0.097
UPDRS III_agility (LLL)	-0.237	0.141	0.102
Visuospatial function	0.065	0.040	0.116
ACE-R	0.012	0.008	0.132
Memory	0.035	0.023	0.143
UPDRS II_writing	0.129	0.090	0.161
UPDRS III_rest tremor persistence	-0.127	0.090	0.169
UPDRS II_hygiene	0.159	0.113	0.170
Attention/orientation	0.068	0.050	0.183
UPDRS III_gait	0.173	0.130	0.192
Rest_tremor	-0.072	0.055	0.201

Parietal

**Variables**	***b*-coefficient**	**SE**	**Unadjusted P-value**
IPAQ (active)	-0.187	0.086	0.038
UPDRS II_gait-balance	-0.115	0.053	0.038
UPDRS III_post_tremor_right	-0.128	0.072	0.085
Work status (retired)	-0.154	0.087	0.087
UPDRS I_halucinations	0.120	0.069	0.090
UPDRS III_rest_tremor (RLL)	0.314	0.181	0.091
Nine-Hole Peg Test (right)	-0.006	0.004	0.094
UPDRS III_rest_tremor (LLL)	0.132	0.077	0.098
UPDRS III_rigidity (RLL)	-0.071	0.044	0.121
UPDRS III_gait_freezing	-0.115	0.073	0.126
UPDRS III_agility (LLL)	0.092	0.061	0.136
Education (incomplete college)	0.330	0.217	0.139
UPDRS IV_fluctuation_complexity	0.049	0.033	0.149
UPDRS III_global_spontaneity	-0.087	0.060	0.158
UPDRS III_neck_rigidity	-0.074	0.051	0.160
Sex (male)	-0.121	0.087	0.174
TMT A	-0.002	0.001	0.183
Postural tremor	-0.056	0.043	0.195
Anxiety (BAI)	0.072	0.056	0.202

ROI, region of interest; SE, standard error; IPAQ, International Physical Activity Questionnaire; UPDRS, Unified Parkinson's Disease Rating Scale (Movement Disease Society); RLL, right lower limb; LLL, left lower limb; TMT A, Trail Making Test - part A; ACE-R, Addenbrooke Cognitive Examination - Revised; BAI, Beck Anxiety Inventory.

Supplementary Table S3. Adjusted multivariate models for frontal, central, and parietal alpha asymmetry in PD.

Variables	β-Coefficient [95% CI]	Standard Error	P-value	Bonferroni correction	Adjusted R^2
FRONTAL					
Diagnosis (years)	-0.042 [-0.079, -0.005]	0.018	0.028	0.140	0.17*
Attention/orientation	0.061 [0.004, 0.118]	0.028	0.037	0.184	
Diagnosis (years)	-0.063 [-0.101, -0.026]	0.018	0.002	0.011	0.29**
UPDRS III_posture	0.136 [0.043, 0.230]	0.045	0.006	0.041	
UPDRS III_rest tremor persistence	-0.111 [-0.210, -0.011]	0.049	0.030	0.211	
CENTRAL					
IPAQ (active)	0.646 [0.343, 0.950]	0.148	0.0001	0.011	0.58***
IPAQ (very active)	0.689 [0.291, 1.086]	0.194	0.001	0.046	
DT-TUG (cost)	0.023 [0.009, 0.037]	0.007	0.002	0.045	
UPDRS II_freezing	0.238 [0.046, 0.430]	0.094	0.017	1.000	
Sex (male)	0.535 [0.237, 0.833]	0.145	0.001	0.025	
PARIETAL					
UPDRS II_gait/balance	-0.156 [-0.260, -0.052]	0.051	0.005	0.037	0.33***
IPAQ (active)	-0.247 [-0.411, -0.083]	0.080	0.005	0.036	

Sex (male)	-0.191 [-0.349, -0.033]	0.077	0.019	0.154

PD, Parkinson’s disease; UPDRS, Unified Parkinson’s Disease Rating Scale (Movement Disease Society); IPAQ, International Physical Activity Questionnaire; DT-TUG (cost), the difference (%) between the single and the dual-task Timed Up and Go tests; * Adjusted for age and sex; ** Adjusted for age, sex and cognition. *** Adjusted for age, cognition, and time since diagnosis.
